# Granulocyte-Colony Stimulating Factor (G-CSF)-Induced Aortitis: A Case Report

**DOI:** 10.7759/cureus.54845

**Published:** 2024-02-24

**Authors:** Masahiro Ito, Masakazu Amari, Akiko Sato, Masahiro Hikichi

**Affiliations:** 1 Breast Surgery, Tohoku Kosai Hospital, Sendai, JPN

**Keywords:** large vessel vasculitis, granulocyte colony-stimulating factor, chemotherapy-related toxicity, aortitis, breast cancer

## Abstract

Pegylated granulocyte colony-stimulating factor (G-CSF), commonly used in chemotherapy-induced neutropenia, has been associated with rare instances of aortitis. This study describes a 67-year-old female patient with estrogen receptor (ER)-positive, human epidermal growth factor receptor-2-positive breast cancer, undergoing chemotherapy with an epirubicin/cyclophosphamide (EC) regimen (epirubicin, cyclophosphamide) and pegylated G-CSF for neutropenia prophylaxis. Post-treatment, she developed symptoms including intermittent fever and severe arthralgia. Laboratory tests revealed an elevated white blood cell count, C-reactive protein levels, and erythrocyte sedimentation rate, while a computed tomography scan showed thickening in the aortic arch and descending aorta. Given the clinical presentation and exclusion of other potential causes, pegylated G-CSF-induced aortitis was suspected. The patient's symptoms improved significantly following the cessation of pegylated G-CSF, aiding in the differentiation from other types of aortitis. This study highlights the importance of considering pegylated G-CSF as a potential cause of aortitis in patients presenting with unexplained symptoms of fever and inflammation after chemotherapy. The rapid improvement upon discontinuation of the drug is a key feature distinguishing it from other aortitis causes. In conclusion, while rare, aortitis should be considered in the differential diagnosis of patients treated with pegylated G-CSF who exhibit relevant clinical symptoms. Early detection and management, including the discontinuation of the causative agent, are crucial for patient recovery and prognosis.

## Introduction

Granulocyte colony-stimulating factor (G-CSF) serves as a vital tool in medical care, particularly for patients undergoing chemotherapy. Its primary function lies in bolstering the bone marrow's capacity to generate neutrophils, essential components of the immune system. By mitigating the risk of neutropenia, a condition characterized by low levels of neutrophils, G-CSF therapy plays a pivotal role in reducing the susceptibility to infections and minimizing the necessity for hospitalization among chemotherapy patients [1]. The use of pegylated G-CSF is expected to increase in the future, and the frequency of aortitis may increase accordingly. Here, we report a case of aortitis induced by pegylated G-CSF administration to prevent neutropenia in a patient with breast cancer. The purpose of this study was to recognize the rare adverse events caused by pegylated G-CSF.

## Case presentation

**Figure 1:**
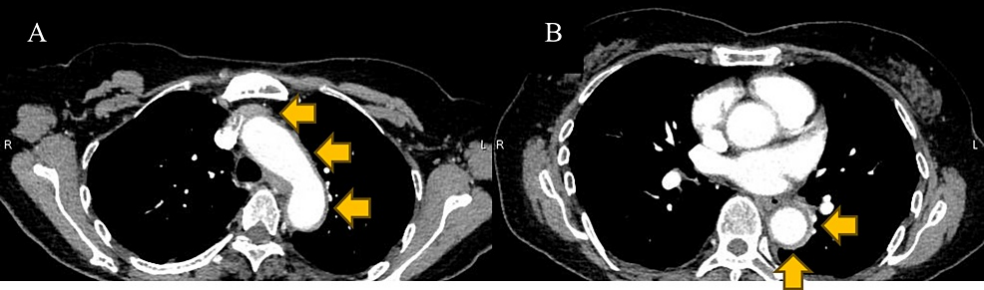
Contrast-enhanced CT scan showing wall thickening of the aortic arch (A) and descending aorta (B) on day 17.

A 67-year-old female presented to our hospital with a history of intermittent fever and severe arthralgia throughout the body. She was diagnosed with estrogen receptor (ER)-positive, human epidermal growth factor receptor-2 (HER2)-positive breast cancer and underwent primary systemic chemotherapy with EC regimen (epirubicin: 90 mg/m^2^, cyclophosphamide: 600 mg/m^2^), together with intravenous dexamethasone (9.9 mg) on day one. She was a hepatitis B virus (HBV) carrier and had a history of mitral regurgitation and glaucoma. The patient was administered oral dexamethasone (6.6 mg) on days two to four and subcutaneously 3.6 mg pegylated G-CSF on day four. On day 11, the patient complained of a high fever of up to 38°C. Since then, she was started on levofloxacin (500 mg/day) prescribed for febrile neutropenia until fever resolved. On day 17, she developed a fever (39°C) again and visited our hospital. On arrival, her physical examination was well except for a high fever. Results of laboratory analysis showed an increased inflammatory response of white blood cell (WBC), 33,800/µL (neutrophil: 91.8%); C-reactive protein concentration (CRP), 19.6 mg/dL; erythrocyte sedimentation rate (ESR), 123 mm/h; and elevation of D-dimmer, 3.3 µg/mL. The polymerase chain reaction test of coronavirus disease 2019 (COVID-19) was negative. Anti-nuclear antibody (ANA), myeloperoxidase-anti-neutrophil cytoplasmic antibody (MPO-ANCA), and serine proteinase 3-anti-neutrophil cytoplasmic antibody (PR3-ANCA) were found to be negative later. Contrast-enhanced computed tomography (CT) scan revealed wall thickening of the aortic arch and descending aorta (Figures 1A, 1B).

**Figure 2:**
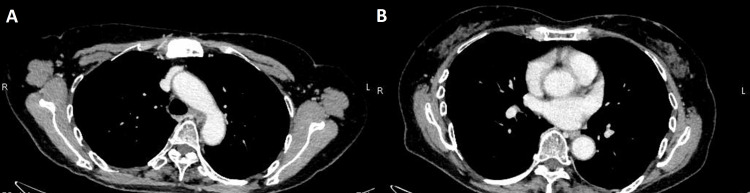
Contrast-enhanced CT six months after discontinuation of pegylated G-CSF showing an improvement in the aortic arch (A) and descending aorta (B). G-CSF: granulocyte colony-stimulating factor

There was no reactivation of HBV, and the echocardiogram showed no verrucae suspicious for infective endocarditis. Although pegylated G-CSF-induced aortitis was suspected based on the CT scan and the absence of findings suggestive of collagen disease, the infection could not be ruled out before culture results proved negative. Therefore, the patient was followed up without the use of steroids. On day 30, the fever resolved, CRP improved, EC therapy was resumed with dose down, and the patient passed without fever. CT after completion of primary systemic chemotherapy performed six months later showed that the thickening of the aortic wall disappeared (Figures 2A, 2B).

## Discussion

The vasculitides are defined by the presence of inflammatory leukocytes in the vessel wall with reactive damage to mural structures. This condition can affect blood vessels of any type, size, or location, leading to a wide range of clinical manifestations. Large-vessel vasculitis (giant cell arteritis, Takayasu arteritis) includes vasculitides that predominantly involve the aorta and its major branches. Medium-vessel vasculitis (polyarteritis nodosa, Kawasaki disease) predominantly involves "medium-sized" arteries, such as those arising from the celiac trunk or the coronary arteries. Small-vessel vasculitis (ANCA-associated vasculitis, etc.) includes vasculitides that predominantly involve small-sized/microscopic, capillaries, and venules [2]. The inflammation in the vessels can lead to changes, such as narrowing, occlusion, aneurysm formation, or rupture, potentially resulting in organ damage or failure due to impaired blood flow [3].

According to the United States Food and Drug Administration Adverse Event Reporting System, the frequency of aortitis after G-CSF administration was 0.0014% [4]. The frequency was higher in Asians, with a G-CSF-induced arteritis frequency of 0.47% in the Japanese adverse event reporting database [4]. When limited to pegylated G-CSF, the frequency was reported to be 0.3% [5]. G-CSF-related aortic symptoms, such as fever, epicardial pain, back pain, constipation, and malaise have been reported, symptoms often appear within 14 days [5,6]. Blood tests usually show elevated markers of inflammation, such as CRP and ESR. Imaging studies, such as CT or magnetic resonance imaging (MRI), can reveal thickening of the aortic wall, which is indicative of inflammation [4,5,7].

In the present study, the differential diagnosis was large-vessel aortitis (giant cell aortitis, Takayasu aortitis). This study did not show the typical features or imaging findings of Takayasu aortitis (absent or weak peripheral pulse, limb claudication, discrepant blood pressure between arms, hypertension, and occlusion or narrowing of large vessels), and Takayasu arteritis tends to be subacute in onset and may last from months to years [8].

Giant cell arteritis, another large vessel vasculitis, was also negative, with no typical symptoms of headache, jaw claudication, or ocular symptoms [3]. ANCA-associated vasculitis was negative in this case, including MPO-ANCA negative and PR3-ANCA negative. Infectious aortitis is another potential differential diagnosis, but it can be ruled out in cases where blood cultures are negative, and there's no evidence of infection around the aorta on imaging. Drug-induced vasculitis has been associated with almost every class of medication and can involve small, medium, and occasionally large arteries [9]. Differentiation from drug-induced arteritis caused by epirubicin or cyclophosphamide was difficult based on clinical symptoms and imaging but was ruled out because the arteritis did not flare up after EC therapy was resumed.

The primary and often initial step in treating G-CSF-associated aortitis is the discontinuation of G-CSF. In many cases, symptoms of G-CSF-associated aortitis start to improve within a few days after discontinuing G-CSF [7,10]. This rapid improvement is a characteristic feature of this condition, distinguishing it from other types of aortitis which might not respond as quickly to changes in treatment [7]. Steroid therapy, such as the use of corticosteroids, is another treatment option. Steroids are known for their potent anti-inflammatory effects and can be effective in reducing inflammation of the aorta. The use of steroids might be considered in more severe cases or when symptoms persist despite the discontinuation of G-CSF [5,11,12]. There are reports that the time from onset to remission was not significantly different between patients who used steroids and those who did not, so it is not clear whether steroids should be used or not [7].

## Conclusions

In conclusion, aortitis is a very rare adverse event caused by pegylated G-CSF. High fever, severe arthralgia, back pain, and elevated CRP or ESR are important differentials, and the possibility of aortitis should be considered after excluding infections and autoimmune diseases. The choice of treatment depends on individual patient factors, including the severity of the condition and the patient's overall health. In many cases, simply discontinuing G-CSF may be sufficient, but steroids can be beneficial in more severe cases or when symptoms persist. Regular monitoring and follow-up are key in both scenarios.
